# Confidence-Aware Gated Multimodal Fusion for Robust Temporal Action Localization in Occluded Environments

**DOI:** 10.3390/s26082454

**Published:** 2026-04-16

**Authors:** Masato Takami, Tomohiro Fukuda

**Affiliations:** Division of Sustainable Energy and Environmental Engineering, Graduate School of Engineering, The University of Osaka, 2-1 Yamadaoka, Suita 565-0871, Osaka, Japan; takami@it.see.eng.osaka-u.ac.jp

**Keywords:** temporal action localization, multimodal learning, pose estimation, robustness, occlusion handling

## Abstract

In industrial environments, robust Temporal Action Localization (TAL) is essential; however, frequent occlusions often compromise the reliability of skeletal data, leading to negative transfer in multimodal fusion. To address this challenge, we propose a Gated Skeleton Refinement Module (Gated SRM), a universal front-end preprocessing module that explicitly incorporates OpenPose confidence scores into the network architecture. By applying these scores as a logarithmic bias within a self-attention mechanism, our method achieves soft suppression—dynamically attenuating the attention weights assigned to unreliable joints—before adaptively fusing the refined skeletal features with RGB representations through a learnable gating network. Extensive experiments on the heavily occluded IKEA ASM dataset demonstrate that our approach effectively prevents the catastrophic accuracy degradation typical of naive and established multimodal fusion strategies, improving the mean Average Precision (mAP) to 21.77%, maintaining parity with the RGB-only baseline while demonstrating superior robustness. Furthermore, the system maintains a practical end-to-end inference speed of approximately 9.2 frames per second (FPS), which is sufficient for monitoring macro-level industrial workflows. By prioritizing confidence-based data selection over data restoration, this sensor-metadata-driven architecture offers a robust and principled approach acting as a critical fail-safe and safety-net for real-world action recognition under occlusion.

## 1. Introduction

In recent years, driven by the advancements in smart cities and Industry 5.0, the significance of video-based Human Action Recognition (HAR) technology has increased rapidly [1]. Within construction and manufacturing sites, there is a surging demand for systems that automatically analyze work activities from surveillance footage to ensure worker safety and optimize operational efficiency [2]. As noted by Luo et al. [3], such industrial environments necessitate wide-area monitoring, which in turn requires robust recognition capabilities from a distance. Furthermore, beyond the mere detection of whether an activity is occurring, Temporal Action Localization (TAL)—which precisely identifies the temporal extent (start and end times) and semantic class of actions of specific tasks—is indispensable for comprehensive and detailed workflow analysis.

The rapid advancement of deep learning techniques has led to a significant leap in the accuracy of HAR [4,5]. Since the introduction of Transformer architecture by Vaswani et al. [6], models based on Attention mechanisms have become predominant in the field of video recognition. For instance, ActionFormer, developed by Zhang et al. [5], successfully achieves high-precision localization of action segments from untrimmed videos by leveraging local self-attention. Moreover, to further improve the robustness of these systems, there is a growing interest in multimodal approaches that combine RGB video data with skeletal features, which represent the geometric structure of the human body [7]. Rehman et al. [1] demonstrated that the integration of RGB and skeletal information allows for recognition accuracy exceeding 98% under clean environmental conditions.

However, applying existing multimodal methods to real-world unconstrained environments, such as construction sites, still poses significant challenges. The core issue lies in the “reliability of sensor data.” As exemplified by the IKEA ASM dataset [8], industrial work sites are characterized by frequent occlusions that compromise skeletal data quality. While skeleton estimation techniques, such as OpenPose [9], offer effective motion descriptions with advantages like privacy protection and viewpoint invariance [10], they are liable to produce low-confidence or erroneous coordinate values for missing joints under occlusive conditions [10,11]. Conventional fusion strategies [12], including the work by Rehman et al. [1], typically perform feature integration under the assumption that all input streams are consistently valid, rendering them vulnerable to such noise contamination. Consequently, there is a substantial risk of “negative transfer,” where degraded skeletal features negate the advantages of the RGB modality, thereby diminishing overall recognition accuracy [13,14].

The objective of this study is to develop a confidence-aware temporal action localization (TAL) system that functions robustly even in real-world environments where occlusions frequently occur. Specifically, we propose a mechanism that directly integrates the confidence scores generated by OpenPose into the attention mechanism of the skeleton feature extraction network. This method functions by adding the logarithm of the confidence score as a bias term to the self-attention layer during the input stage of ActionFormer [5]. This enables the model to adaptively decay the weights of joint information, whose reliability is compromised by occlusion. This mechanism effectively prevents negative transfer (adverse effects on RGB features) during multimodal fusion. The result is flexible and seamless integration based on data quality. Specifically, it maximizes the usefulness of geometric features when they are accurate and minimizes their impact when they are inaccurate. We evaluated the effectiveness of this method using the IKEA ASM dataset [8], characterized by severe occlusions, and demonstrated its superiority over conventional methods.

The primary contributions of this work are summarized as follows:

- We propose a confidence-biased self-attention mechanism that incorporates log-transformed pose estimation confidence scores as bias terms in the attention weight computation, achieving continuous soft suppression of unreliable skeletal features.
- We develop a Gated Skeleton Refinement Module (Gated SRM) that purifies skeletal information prior to feature fusion via a learnable gating network, designed to prevent the fused representation from degrading below the RGB-only baseline even under heavy occlusion.
- We validate the proposed method on both the standard THUMOS14 benchmark and the heavily occluded IKEA ASM dataset, demonstrating consistent improvements over RGB-only baselines and conventional fusion approaches.

The remainder of this paper is organized as follows. Section 2 provides an overview of related research on temporal action localization (TAL) and sensor fusion. Section 3 details the architecture of the proposed system. Section 4 describes the verification process using a prototype system, and Section 5 presents the verification test. Finally, Section 6 offers insights derived from the findings, and Section 7 concludes the paper.

## 2. Related Work

### 2.1. Video-Based Temporal Action Localization

#### 2.1.1. Evolution from Video Classification to Temporal Action Localization

Research in HAR within the field of computer vision has undergone significant advancements over the past several decades. In early studies, the predominant task was Video Classification, which involves assigning a single action label—such as “walking,” “running,” or “waving”—to short, trimmed video clips [15,16]. While these methods achieved certain successes under constrained computational resources, they often lacked robustness against complex backgrounds and illumination variations. Furthermore, they faced inherent limitations in capturing high-level semantic features [4,15].

The emergence of deep learning fundamentally transformed this landscape. The Two-Stream Convolutional Network, proposed by Simonyan et al. [17], introduced a seminal architecture that processes spatial appearance information (RGB images) and temporal motion information (optical flow) through separate convolutional neural networks (CNNs) before eventually fusing them. This approach mimics the human cognitive process of integrating visual and motion information in the brain, leading to a dramatic improvement in the accuracy of Action Recognition. Furthermore, I3D, developed by Carreira et al. [18], enabled the transfer of knowledge from large-scale image datasets to the video domain by “inflating” 2D kernels pre-trained on ImageNet along the temporal dimension. Consequently, this model has become the de facto standard for large-scale video datasets, such as Kinetics-400 [18,19].

However, in real-world deployment scenarios, videos are typically not pre-trimmed; instead, they are provided as long, untrimmed streams. Given this context, the primary research focus has shifted from simple classification to TAL [20]. This task involves identifying both the start and end timestamps of action instances within untrimmed videos while simultaneously identifying their respective action classes.

#### 2.1.2. Introduction of the Transformer Architecture and the Development of ActionFormer

The most significant breakthrough in recent research on TAL is the introduction of Transformer architecture, which has achieved overwhelming success in the field of Natural Language Processing (NLP) [6]. The self-attention mechanism, which serves as the core of the Transformer, allows for the direct modeling of relationships between elements at arbitrary positions within a sequence. Consequently, this mechanism enables the effective utilization of contextual information surrounding action segments [4,5].

ActionFormer, presented by Zhang et al. [5], is a prominent model that optimizes the Transformer architecture specifically for TAL tasks. It has achieved state-of-the-art (SOTA) performance on major benchmarks, such as THUMOS14 and ActivityNet-1.3, significantly outperforming conventional methods [21,22].

Following the success of ActionFormer, several variants offering higher accuracy and efficiency have been proposed in succession. TriDet [23] introduced the concept of “trigger detection” to address the ambiguity of action boundaries, leading to a significant improvement in boundary estimation accuracy. In addition, LGAFormer [24] attains more robust feature representations by integrating local and global attention mechanisms.

However, these SOTA methods primarily focus on structural improvements to the detection head, the refinement of boundary regression, and the optimization of computational efficiency. These approaches inherently assume that the RGB and skeletal features are “reliable” (i.e., clean and noise-free). Consequently, there remains a significant research gap regarding effective countermeasures for scenarios where the reliability of the input data itself is compromised by occlusion or fluctuations in lighting—conditions that directly challenge the robustness of the localization process.

#### 2.1.3. Next-Generation Foundational Technology: State Space Models (SSM) and Mamba

To address the computational cost issues of Transformer—specifically the quadratic increase in computational complexity relative to sequence length—State Space Models (SSMs), particularly the Mamba architecture, have recently garnered significant attention [25]. In the field of video recognition, pioneering efforts such as ActionMamba [26] have begun to emerge. ActionMamba leverages the “Selective Scan” capability of Mamba blocks to incorporate a mechanism that retains essential action information within video sequences while discarding irrelevant background noise [26]. This approach suggests the potential to resolve both the limitations of GCN-based methods in global context understanding and the computational overhead inherent in Transformer [19,26]. However, SSM-based TAL methods are still in their infancy; their integration with established detection heads and post-processing pipelines, such as those employed by ActionFormer, has not yet been sufficiently validated. Furthermore, since SSMs are specialized for one-dimensional sequence modeling, the efficient embedding of spatial structural information, such as image or skeleton data, remains an ongoing challenge [10].

### 2.2. Action Recognition Using Skeleton Data

#### 2.2.1. Characteristics and Advantages of Skeleton Data

The utilization of skeleton information (Skeleton Data) has established an indispensable status in the field of HAR as a modality that complements the inherent limitations of RGB video [1,10,27]. Skeleton data is represented as a temporal sequence of 2D or 3D coordinates of primary human joint points (keypoints). This representation is of paramount importance from the perspectives of privacy preservation, data efficiency, and background invariance [10,27,28].

#### 2.2.2. Evolution of Graph Convolutional Networks (GCN) and the “Reliability Gap” in Pose Estimation

In skeleton-based action recognition, modeling the human body structure as a graph has become a standard approach [29]. Spatial–Temporal Graph Convolutional Networks (ST-GCNs) were introduced by Yan et al. [29] in 2018, representing a seminal work in this field. ST-GCNs construct a spatial–temporal graph by integrating a spatial graph structure—where joints are defined as nodes (vertices) and bones as edges—with temporal edges that connect identical joints across successive frames. By applying graph convolution operations to this structure, the model effectively extracts discriminative features of human actions.

Since the inception of ST-GCNs, numerous refined methods have been proposed to enhance the representational capacity of graph structures [10,26,30,31]. While skeleton-based approaches are theoretically powerful, their performance is heavily reliant on the quality of the input skeleton data. In real-world scenarios, skeleton features are typically extracted using image-based pose estimation algorithms such as OpenPose [9]; however, these estimators are not infallible. Specifically, the accuracy of pose estimation degrades significantly in the presence of occlusion (where objects hide body parts) or self-occlusion (where body parts overlap), leading to unreliable joint coordinates [10,13].

State-of-the-art pose estimators, such as OpenPose [9], output a confidence score representing the certainty of the estimation alongside the spatial coordinates (x,y) for each joint. These confidence scores can serve as a critical indicator for determining whether a joint is visible or occluded [9,10,14]. However, many existing skeleton-based action recognition methods [29] typically discard these confidence scores at the input stage, relying solely on coordinate information (x,y) as geometric features [32]. This convention treats unreliable, noisy data as equivalent to reliable, accurate data. Consequently, this causes the model to learn erroneous motion patterns when occlusion occurs, resulting in a degradation of recognition accuracy.

#### 2.2.3. Existing Approaches and Their Limitations

To address the noise inherent in skeleton data, several prior studies have focused on “data restoration.” Song et al. [13] proposed the “Richly Activated GCN,” designed to extract robust features even from incomplete skeleton data, attempting to compensate for missing joint information through multi-stream fusion. Similarly, Yoon et al. [11] developed a method to predict and impute currently missing joints based on information from preceding frames.

These approaches are fundamentally aimed at approximating the Ground Truth (GT) of skeleton data. However, in scenarios characterized by severe occlusion, such as construction sites or complex working environments, the loss of information is often too extensive to allow for accurate restoration or repair [3,10]. Introducing inaccurately reconstructed skeleton data—effectively fabricated joint coordinates—into the model poses a significant risk of inducing overconfidence in the predictions [33]. We define the term “Reliability Gap” as the discrepancy between the apparent format of the data output by sensors (or estimators) and the actual reliability of its content. Existing restoration-based methods do not effectively bridge this gap; instead, they risk introducing additional noise in their attempt to fill it. Consequently, they remain problematic for real-world applications where safety and reliability are paramount [3,33].

### 2.3. Multimodal Sensor Fusion and Reliability

#### 2.3.1. Fusion Strategies and the Manifestation of the Reliability Gap

To overcome the inherent limitations of single modalities (such as RGB-only or skeleton-only approaches), multimodal sensor fusion—which integrates information from multiple sensors—is viewed as a promising solution. Rehman et al. [1] reported that integrating RGB video with skeleton tracking data can significantly improve the accuracy of HAR. Furthermore, more sophisticated cross-modal fusion and temporal localization techniques have emerged to exploit structural cues. For instance, Zhang et al. [34] proposed the Pose-Guided Video Transformer (PGVT), which effectively incorporates sparse high-level body joints into vision transformers for fine-grained action recognition. In the context of temporal action localization, Liu et al. [35] introduced BRTAL, utilizing an offset-driven diffusion model to progressively refine action boundaries from a local perspective.

While multimodal integration generally improves average performance [1,7], simple feature concatenation relies on the implicit assumption that all modalities maintain constant reliability [33,36]. In real-world environments where sensor quality fluctuates dynamically, this assumption leads to the negative transfer problem defined in Section 1—a direct manifestation of the Reliability Gap (defined in Section 2.2.3) at the system level. This poses an unacceptable risk in safety-critical applications [3,12,31].

#### 2.3.2. Reliability-Aware Learning

To address this challenge, some studies have employed approaches that utilize attention mechanisms for feature weighting [7,37,38]. Mechanisms such as the cross-attention found in Transformers are capable of learning cross-modal correlations and prioritizing salient information. However, since attention mechanisms are fundamentally trained to identify “discriminative features” relevant to the task [6], they run the risk of assigning high attention weights even to noise if it appears distinctive. In other words, relying solely on internal attention mechanisms within neural networks may be insufficient to accurately assess the physical signal integrity of the sensors.

In contrast, the confidence scores inherently output by the pose estimator serve as objective metadata representing the intrinsic quality of the sensor data itself. However, to the best of our knowledge, research that utilizes this confidence score as an explicit gating signal to dynamically modulate feature fusion when integrating skeleton recognition with state-of-the-art TAL models, such as ActionFormer [5], remains extremely limited [10,19]. Most existing studies relegate confidence scores to simple thresholding operations (e.g., discarding low-confidence joints) and fail to incorporate them directly into the neural network’s training process as a fully differentiable gating mechanism [10,39].

### 2.4. Research Positioning and Contributions

Based on the survey of related work presented above, three critical unresolved issues (Research Gaps) have emerged within the current landscape of TAL and multimodal recognition:

- Limitations of RGB Dependence: While SOTA models such as ActionFormer [5] exhibit powerful feature extraction capabilities, they remain susceptible to visual noise and occlusion, often incurring high computational costs.
- Vulnerability of Skeleton Features: Skeleton-based models like ST-GCNs [29] offer a lightweight alternative; however, they are extremely fragile against data missingness, and noise caused by occlusion, lacking reliability when used in isolation.
- Neglect of Dynamic Reliability: Existing multimodal fusion approaches do not explicitly account for dynamic quality fluctuations (i.e., confidence gaps) across sensors, failing to fully eliminate the adverse effects of low-quality data.

To address these challenges, we present a novel sensor fusion framework leveraging Confidence-Weighted Skeleton Features. The uniqueness and contributions of this research are clearly defined in the following aspects:

- Confidence-Driven Data Selection vs. Data Restoration: Restoration approaches [11,13] attempt to reconstruct missing joint coordinates, risking hallucinated predictions under severe occlusion. In contrast, our method bypasses reconstruction entirely by utilizing confidence scores as continuous gating signals, representing a fundamental paradigm shift from “data repair” to “data selection.”
- Explicit Confidence Integration: While conventional attention-based fusion learns “what to look at,” our approach explicitly guides the model on “what to trust” based on sensor metadata. This prevents the neural network from erroneously learning from noise.
- Validation under Realistic Occlusion Conditions: Unlike prior TAL studies that primarily evaluate on cleanly captured benchmarks [5,23,24], we validate on the IKEA ASM dataset [8], whose heavy occlusion characteristics (detailed in Section 4.2) closely approximate industrial deployment conditions.

## 3. Proposed Methods

### 3.1. System Overview

**Figure 1:**
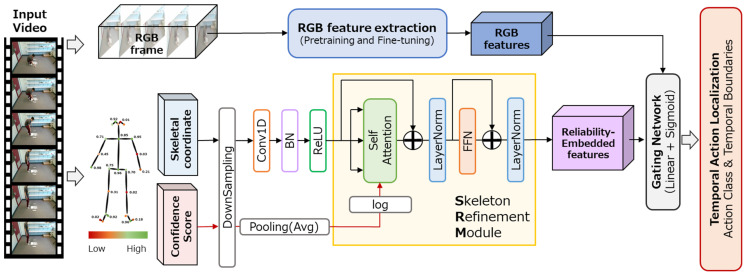
Overview of the temporal action localization system incorporating the proposed Gated Skeleton Refinement Module (Gated SRM). The system adaptively fuses RGB features extracted from the input video with skeletal features (Reliability-Embedded features), which are refined through a self-attention mechanism using confidence scores. This fusion is performed via a learnable gating mechanism (Gating Network). The resulting fused features are dimensionally compatible with the base TAL model, requiring no architectural modifications downstream.

In this study, we present the Gated Skeleton Refinement Module (Gated SRM), designed to effectively integrate skeletal features with RGB features for TAL. The overall architecture of the system is illustrated in Figure 1.

The proposed method is a front-end module integrated into an existing Transformer-based TAL model. It takes pre-extracted RGB features and skeletal coordinates as input and generates fused features by adaptively controlling the contribution of skeletal information through a learnable gating mechanism. Because the generated fused features maintain the same dimensionality as the original RGB features, the design allows the system to leverage the benefits of skeletal information without requiring any structural modifications to the subsequent TAL model architecture.

The overall architecture of the proposed method consists of three stages: (1) the Skeleton Refinement Module (SRM), which refines skeletal features using a confidence-biased self-attention mechanism; (2) dimensional transformation via a projection layer; and (3) adaptive fusion with RGB features through a gating mechanism.

### 3.2. Skeleton Refinement Module (SRM)

The SRM is a module designed to refine raw skeletal coordinate sequences along with the temporal dimension and extract high-level skeletal features. Since coordinate values obtained from existing pose estimation methods contain variances in reliability caused by occlusion and motion blur, this module introduces Confidence-Biased Self-Attention, which incorporates a confidence bias into the attention scores.

#### 3.2.1. Temporal CNN Projection

First, we apply a projection layer—consisting of a 1D convolution with a kernel size of 3, batch normalization, and ReLU activation—to the input skeletal coordinates $S\in R^{B\times 50\times T}$ to obtain the embedded features $X\in R^{B\times d_{s}\times T}$ ($d_{s}=512$). This preprocessing step captures local temporal context while simultaneously unifying the feature dimensionality with the RGB embedding layer of the base model.

#### 3.2.2. Confidence-Biased Multi-Head Self-Attention

We apply Multi-Head Self-Attention to the obtained embedded features X. Following the standard Transformer formulation, the attention score (logit) $e_{ij}$ between a query $q_{i}$ and a key $k_{j}$ is typically computed as scaled dot-product attention (Equation (1)):

(1) $$e_{ij}=\frac{q_{i}{k_{j}}^{T}}{\sqrt{d_{k}}}$$

where $d_{k}$ is the dimension of the key heads. To explicitly integrate the reliability of skeletal estimates into this mechanism, we inject structural information by adding a learnable bias term to the attention logits. In our framework, we incorporate a bias term derived from the skeletal confidence scores $c\in R^{B\times 1\times T}$ into the standard attention score on the Key side.

Here, the per-frame confidence score c is obtained by computing the arithmetic mean of the confidence scores across all 25 joints at each time step. The modified attention score ${e^{\prime }}_{ij}$ is defined as Equation (2):

(2) $${e^{\prime }}_{ij}=e_{ij}+\;log(c_{j}+\varepsilon )$$

where $\varepsilon =10^{-6}$ is a small constant introduced for numerical stability, and $c_{j}$ represents the confidence score of the key frame at time step j.

Notably, the logarithmic transformation enables the bias to function as multiplicative weight control within the Softmax function. By substituting $e_{ij}$ into the Softmax operation, the attention weight $\alpha_{ij}$ is derived as Equation (3):

(3) $$\alpha_{ij}=\frac{exp({e^{\prime }}_{ij})}{\sum_{n}exp({e^{\prime }}_{in})}=\frac{exp(e_{ij}+\;log(c_{j}))}{\sum_{n}exp(e_{in}+\;log(c_{n}))}=\frac{exp(e_{ij})\cdot c_{j}}{\sum_{n}exp(e_{in})\cdot c_{n}}$$

As shown in this derivation, the confidence scores $c_{j}$ directly scale the exponential of the raw attention score. This mechanism ensures that features with high confidence ($c_{j}\approx 1)$ retain their original affinity, while those with low confidence ($c_{j}\to 0)$ are exponentially suppressed toward zero, effectively filtering out unreliable skeletal information before feature aggregation.

Finally, the output of the attention head is computed as Equation (4):

(4) $$Attention(Q,K,V)=Softmax(\frac{QK^{T}}{\sqrt{d_{k}}}+log(C+\varepsilon ))V$$

where C is the matrix of confidence scores broadcast across the query dimension.

Following the attention output, we apply residual connections and Layer Normalization, followed by a Feed-Forward Network (FFN) with an expansion ratio of 4. After a subsequent round of residual connections and Layer Normalization, we obtain the refined skeletal features $X_{refined}\in R^{B\times d_{s}\times T}$.

### 3.3. Gated Fusion Mechanism

Rather than employing simple concatenation or addition, we introduce a learnable gating mechanism to fuse the refined skeletal features $X_{refined}\in R^{B\times 512\times T}$ with the RGB features $F_{RGB}\in R^{B\times 1024\times T}$. This mechanism enables the model to adaptively adjust the contribution of skeletal information at each time step and channel, depending on the specific video content and action types.

First, we project the skeletal features into the same dimensionality as the RGB features (Equation (5)).

(5) $$F_{skel}=ReLU(BN(W_{p}\times X_{refined}))\in R^{B\times 1024\times T}$$

where $W_{p}$ denotes the weights of a 1D convolution with a kernel size of 1.

Next, we concatenate the RGB features and the projected skeletal features to compute per-channel gate values (Equation (6)).

(6) $$g=\sigma (W_{g}\times \left[F_{RGB};F_{skel}\right])\in R^{B\times 1024\times T}$$

In this equation, [·;·] represents channel-wise concatenation (yielding 2048 dimensions), $W_{g}$ is a 1D convolution layer that maps the 2048-dimensional vector to 1024 dimensions, and σ denotes the Sigmoid activation function.

The final fused features are calculated as a weighted addition of the skeletal features, modulated by the gate values (Equation (7)).

(7) $$F_{fused}=F_{RGB}+g⊙F_{skel}$$

where ⊙ denotes the element-wise product.

To ensure stability during the initial stages of gate network training, we initialize the weights of the gating layer to zero and the initial bias to $b_{init}=-2.0$. Consequently, the initial gate output is σ(−2.0)≈0.12, which ensures that the fused features are approximately equal to the RGB feature ($F_{fused}\approx F_{RGB})$ at the onset of training. The empirical validation of this specific initialization value, compared to alternative strategies, is detailed in Section 5.6. This initialization strategy offers the following advantages:

- Preservation of Pre-trained Representations: It prevents the destruction of the pre-trained RGB backbone’s representations during the early phases of training.
- Progressive Learning: It enables a progressive learning process where the contribution of skeletal information increases gradually over time.
- Robustness as a Safety Valve: It functions as a safeguard that automatically suppresses the gate output when the quality of skeletal features is compromised, such as in environments with heavy occlusion.

### 3.4. Integration with Base Model

The proposed Gated SRM functions as a front-end preprocessing module integrated into the base TAL model. Specifically, for each video, the module receives RGB features $F_{RGB}\in R^{1024\times T}$, skeletal coordinates $S\in R^{50\times T}$, and confidence scores $c\in R^{1\times T}$ as inputs. After generating the fused features $F_{fused}\in R^{1024\times T}$ through the Gated SRM, these features are fed into the base model in place of the original RGB features.

Because the dimensionality of the fused features remains identical to that of the RGB features (1024 dimensions), no structural or parametric modifications are required for the base model components, such as the backbone network, Feature Pyramid Network (FPN), or the classification and regression heads. This architectural design ensures that the pre-trained weights of the base model can be utilized directly, facilitating either the isolated fine-tuning of the proposed module or comprehensive end-to-end training of the entire system.

## 4. Implementation of Prototype System

### 4.1. Development Environment

The development and evaluation of the prototype system were conducted on a workstation running Ubuntu 24.04 LTS. The hardware configuration utilized an Intel Core i7-8700 CPU and an NVIDIA GeForce GTX 1080 GPU. Regarding the software environment, we employed Python 3.9 and the PyTorch 2.5.1 framework, using OpenCV 4.12.0 for video input/output and preprocessing. For reproducibility, the complete source code and pre-trained weights are provided in the Supplementary Materials.

In this implementation, we prioritized a lightweight design with low computational overhead to facilitate potential deployment on edge devices. Specifically, the skeletal processing stream is engineered to maintain a low computational load, ensuring high adaptability for real-time processing applications.

### 4.2. Datasets

For the evaluation, we utilize the THUMOS14 dataset [40], a standard benchmark in the field of action recognition, and the IKEA ASM dataset [8], which simulates real-world environments characterized by frequent occlusions.

**Figure 2:**
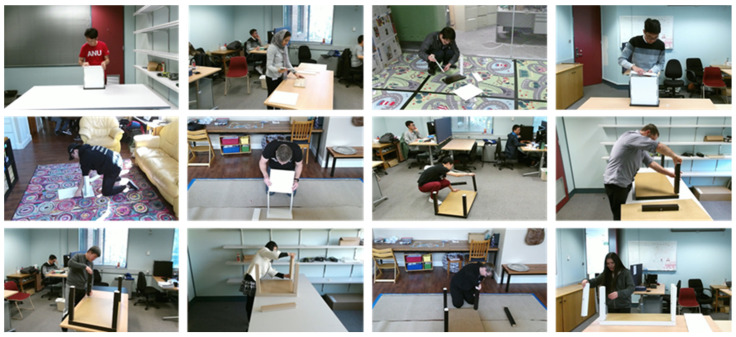
Sample frames from the IKEA ASM dataset. This dataset captures furniture assembly tasks where self-occlusion and occlusion by objects occur frequently. It comprises 32 categories of atomic actions, such as “attaching legs” and “flipping the tabletop,” exhibiting characteristics highly representative of real-world environments.

- THUMOS14 dataset [40]: This is a large-scale dataset comprising untrimmed videos of sports activities collected from YouTube. It contains 20 action classes, including “Long Jump” and “Cricket Bowling.” While the dataset exhibits significant camera motion and diverse backgrounds, the subjects are typically large and clearly visible within the frames, with a relatively low frequency of occlusions. To ensure a fair comparison with existing studies [5,40,41,42,43,44], we utilize the standard subset consisting of the validation set (200 videos) and the test set (213 videos).
  Although THUMOS14 exhibits relatively low occlusion frequency, we include it as an evaluation benchmark for two reasons: (1) to verify that the proposed method does not degrade performance on well-established benchmarks, and (2) to demonstrate that the gating mechanism appropriately increases skeletal contribution when skeleton quality is high, thereby improving upon the RGB-only baseline.
- IKEA ASM dataset [8]: This dataset consists of 371 video recordings capturing the assembly of various furniture items, such as tables and shelves. It contains a total of approximately 35 h of footage. The average duration per video is about six minutes. The dataset is densely annotated, yielding approximately 31,000 action instances (clips) across the entire dataset.
  It comprises 33 officially defined atomic action classes (typically utilized as 32 foreground action classes plus one background class in Temporal Action Localization tasks). These classes are structured as verb–object pairs (e.g., “attaching legs” and “spinning in”). Sample frames from the IKEA ASM dataset are illustrated in Figure 2. The defining characteristic of this dataset is the frequent occurrence of severe self-occlusion and occlusion by objects, resulting from workers bending forward deeply or being obscured by furniture components. These attributes closely mirror the conditions of real-world environments, such as construction sites and factories, making it an ideal testbed for evaluating the robustness of our proposed method.

For the validation of our research, we have adopted the “Environment-based Train/Test Split” protocol established in the official dataset publication. This strategy is designed to prevent overfitting that could occur if identical subjects or rooms (environments) appear in both the training and testing data. Specifically, among the five assembly environments, we strictly isolate Environments 1 and 2 (the family room and the office) to serve as the test set (117 videos). Consequently, we utilize the remaining environments as the training set (254 videos).

### 4.3. Base Model: ActionFormer

We adopt ActionFormer [5] as the base model for TAL. ActionFormer is a single-stage detection model that applies a Transformer-based backbone equipped with a Feature Pyramid Network (FPN) to pre-extracted video features, simultaneously performing action classification and temporal boundary regression at each time step.

Table 1 presents the key hyperparameters of the base model. The backbone architecture consists of a convolutional Transformer (convTransformer) with a layer configuration of (2, 2, 5) and a scale factor of 2. Both the embedding dimension and the FPN dimension are set to 512, with the number of attention heads set to 4 and the local attention window size to 19. The detection head comprises three layers with a hidden dimension of 512, and the regression ranges are defined as [0, 4], [4, 8], [8, 16], [16, 32], [32, 64], and [64, 10,000]. During inference, we apply Soft-NMS with an IoU threshold of 0.4 and retain the top 200 predictions per video, following the standard ActionFormer configuration [5].

### 4.4. Implementation of the SRM

To improve recognition accuracy in scenarios with frequent occlusion, we design a SRM that leverages human skeletal features alongside RGB features. The SRM comprises the following three components:

- Temporal CNN Projection Layer: We map the 50-dimensional input skeleton coordinates (25 joints × 2 coordinates) into a 512-dimensional feature space using a 1D Convolutional Neural Network (1D-CNN) with a kernel size of 3 and a padding of 1, followed by Batch Normalization and a ReLU activation function.
- Confidence-Biased Multi-Head Self-Attention: We utilize 8 heads (with a dimension of 64 each). A confidence bias term $log(c+\varepsilon )(\varepsilon =10^{-6})$ is applied to the Key side during the attention score calculation.
- Feed-Forward Network (FFN): This component consists of two 1D convolutional layers (512 → 2048 → 512) accompanied by ReLU and dropout (with a rate of 0.1).

For both datasets, skeletal coordinates and confidence scores were extracted using OpenPose [9] with the BODY_25 model (25 joints). To quantitatively validate the assumption that these confidence scores reliably reflect the spatial accuracy of the estimated joints under realistic occlusions, we conducted an empirical error analysis measuring the spatial jitter. The detailed results, demonstrating a significant negative correlation between confidence and spatial error, are provided in Appendix A. While 3D skeletal representations or depth-based enhanced features can theoretically provide richer spatial context, we deliberately adopted the 50-dimensional 2D skeletal representation (25 joints × 2 coordinates) to ensure practical applicability in real-world industrial deployments. As we discuss later in Section 5.2.2, relying on 3D data typically necessitates specialized equipment such as depth sensors, which are often impractical or prohibitively expensive for widespread factory surveillance. By operating exclusively on 2D coordinates extracted from standard RGB video, our approach maintains computational efficiency and offers a highly cost-effective solution. For THUMOS14, OpenPose was applied to the same frames used for I3D feature extraction, and in scenes with multiple people, the person with the highest average confidence score was selected, and frames with no detected persons were assigned zero-filled coordinates with confidence scores of zero. For the publicly available I3D features, the published 2048-dimensional features were used, which were pre-trained on Kinetics-400, following the standard protocol [5]. For IKEA ASM, the I3D model (RGB stream only, without optical flow) was fine-tuned on the training split for 20 epochs with a learning rate of 0.01, and features with 1024 dimensions were extracted by setting a temporal interval of 4 frames.

### 4.5. Fusion Strategies

To integrate RGB features and skeletal features, we implement two strategies: a baseline “Naive Concatenation” and our developed “Gated Fusion”.

- Naive Concatenation: We simply concatenate the 512-dimensional output of the SRM and the RGB features (1024 or 2048 dimensions) along the channel dimension, feeding the result directly into the backbone. Consequently, this approach requires modifying the input dimension of the backbone (e.g., from 1024 to 1536 for the IKEA ASM dataset).
- Gated Fusion (Proposed Method): This approach maintains the original input dimension of the backbone, enabling the direct utilization of pre-training weights. It operates in the following three steps:
  1. We map the SRM output to the same dimension as the RGB features via a projection layer (e.g., a 1 × 1 convolution).
  2. We concatenate the RGB features with the projected skeletal features to generate gate values in the range of [0, 1] using a 1 × 1 convolution and a sigmoid function.
  3. We compute the element-wise sum of the RGB features and the gate-modulated skeletal features as the final fused representation.

The gate initialization follows the strategy described in Section 3.3 (weights = 0, bias = −2.0). The detailed architectural configurations and hyperparameters for both the Skeleton Refinement Module (SRM) and the Gated Fusion Module are summarized in Table 2.

### 4.6. Training Protocol

All models are trained using the AdamW optimizer with a learning rate of ${1\times 10}^{-4}$ and a weight decay of 0.05. The batch size is set to 2, and the training is conducted for a total of 55 epochs, including 5 warmup epochs. For data augmentation, we employ random cropping with a truncation threshold of 0.5 and a crop ratio of [0.9, 1.0], while applying gradient clipping with an L2 norm of 1.0. We utilize an Exponential Moving Average (EMA) for evaluation, and the model achieving the highest mean Average Precision (mAP) during validation, which is performed every 5 epochs, is selected as the final model.

## 5. Verification Test

In this section, we assess the effectiveness of the proposed Gated SRM through both quantitative and qualitative analyses, using two datasets described in Section 4.2: THUMOS14 and IKEA ASM. Furthermore, we extend our analysis to demonstrate the module’s generalizability to other base architectures and present an ablation study on the gate bias initialization to empirically validate our design choices.

### 5.1. Experimental Setup

#### 5.1.1. Baselines

In this study, to rigorously evaluate the effectiveness of our approach, we compare the proposed method against the RGB-only baseline, established multimodal fusion strategies, and our ablation configurations:

1. RGB-only baseline (#1): ActionFormer using only RGB features.
2. Naive Concatenation (#2): Skeleton features projected by CNN and combined with RGB features along the channel dimension.
3. SRM + Concatenation (#3): Skeleton features refined by SRM and simply concatenated with RGB features.
4. Gated Fusion without SRM (#4): Skeleton features projected by CNN and fused through our gating mechanism without confidence bias.
5. Cross-Attention Fusion (#5): An established attention-based integration technique utilizing standard Multi-Head Cross-Attention, where RGB features serve as queries and skeleton features as keys and values.
6. Late Fusion (#6): An ensemble strategy where predictions from independently trained RGB and Skeleton models are combined. The proposal scores are weighted equally (0.5/0.5) and integrated using Soft-NMS (IoU threshold 0.1) to suppress duplicates.
7. Proposed method (Gated SRM): The full pipeline proposed in this work.

#### 5.1.2. Evaluation Metrics

This section outlines the evaluation metrics utilized to verify the proposed method. To conduct a multifaceted evaluation of performance in the TAL task, we employ two distinct metrics: mean Average Precision (mAP) and the Boundary-F1 score.

- mean Average Precision (mAP)

mAP is a standard evaluation metric widely adopted in TAL [40,45,46,47,48]. Specifically, we calculated mAP values at various temporal Intersection over Union (t-IoU) thresholds and utilized their average as the final evaluation metric. The t-IoU was computed according to Equation (8).

(8) $$tIoU=\frac{prediction\cap GT}{prediction\cup GT}$$

where prediction indicates the proposed model’s detected actions, and GT refers to the pre-annotated ground-truth activity segments.

In this study, the mAP is calculated according to the following procedure:

1. Set t-IoU Thresholds: We establish five levels of temporal Intersection over Union (t-IoU) thresholds: 0.3, 0.4, 0.5, 0.6, and 0.7.
2. Determine True Positives (TP): For each detected action instance, the prediction is classified as a True Positive (TP) if its t-IoU with the ground truth segment is equal to or greater than the specified threshold and the action class matches correctly.
3. Rank Predictions: For each action class, all predictions are ranked in descending order of their confidence scores to compute Precision and Recall.
4. Calculate Average Precision (AP): The Average Precision (AP) is determined by calculating the area under the Precision–Recall curve for each class.
5. Compute mAP: Finally, the mAP is obtained by averaging the AP values across all action classes.

The mAP is computed using Equation (9).

(9) $$mAP=\frac{\sum_{j=1}^{C}{AP}_{\left(j\right)}}{C}$$

where C is the number of action categories.

In this study, we conduct a multifaceted performance evaluation by employing five distinct IoU thresholds. As a final comprehensive assessment, we calculate the average of the mAP values across these five thresholds to serve as the overall performance metric for the system. Furthermore, the Average Precision (AP) for each individual action class obtained during the mAP calculation process is used for an in-depth per-class performance analysis.

- Boundary-F1 score

The Boundary-F1 score is an evaluation metric specifically designed to assess the precision of identifying action boundaries, namely, the start and end points of an action. While mAP evaluates the overall overlap of action segments, the Boundary-F1 score directly measures the temporal accuracy of the boundary points themselves [21,47]. In the calculation of the Boundary-F1 score, the success of a prediction is determined by whether a predicted boundary point falls within a specific time window (tolerance τ) of the ground truth boundary. In this study, we established three levels of tolerance: τ = ±0.5 s, τ = ±1.0 s, and τ = ±2.0 s. We computed the Boundary-F1 score in this research according to the following procedure.

1. A predicted boundary point is classified as a True Positive (TP) if it falls within ± τ seconds of a ground truth (GT) boundary point and the action class matches.
2. A prediction is classified as a False Positive (FP) if no ground truth exists within the tolerance window or if the prediction is redundant.
3. A ground truth boundary is classified as a False Negative (FN) if no predicted boundary point exists within the specified tolerance.
4. Precision and Recall are calculated using Equation (10) and Equation (11), respectively.
  (10) $$Precision=\frac{TP}{\left(TP+FP\right)}$$
  (11) $$\begin{array}{c} Recall=\frac{TP}{\left(TP+FN\right)} \end{array}$$
5. The F1 score is then calculated using Equation (12).
  (12) $$\begin{array}{c} F1=2\times \frac{\left(Precision\times Recall\right)}{\left(Precision+Recall\right)} \end{array}$$

### 5.2. Quantitative Results

#### 5.2.1. Comparison of THUMOS14

Table 3 presents the mAP comparison results on the THUMOS14 dataset. ActionFormer, serving as the baseline with only RGB input, achieved a mAP of 65.87%. In contrast, a degradation in performance was observed when skeletal data were naively integrated. Specifically, simple concatenation following CNN projection resulted in a mAP of 63.22%, and even with the addition of a SRM, the performance remained at 63.44%; both configurations underperformed relative to the RGB-only baseline. This phenomenon represents a typical case of “negative transfer,” where noisy skeletal information is integrated without selective processing, thereby hindering the representation of effective RGB features.

Conversely, fusion methods incorporating a gating mechanism demonstrate performance improvements. The combination of CNN projection and gated fusion recovered the mAP to 64.89%. When comparing with established fusion strategies, Cross-Attention Fusion (#5) achieved a mAP of 64.71%, while Late Fusion (#6) recorded 65.32%. Neither was able to surpass the RGB-only baseline. Furthermore, our proposed approach, “SRM + gated fusion,” achieved the highest mAP of 66.31%, performing comparably to the baseline.

Furthermore, regarding the Boundary F1 score—an indicator of action boundary detection performance—the proposed method maintained accuracy levels comparable to the RGB-only baseline. Under the condition of a tolerance threshold τ = ±0.5 s, the proposed method recorded a score of 0.5665 compared to 0.5659 for the RGB-only configuration. Notably, while Late Fusion maintained competitive mAP, it exhibited a severe degradation in the Boundary-F1 score (0.2557). This suggests that merging temporally imprecise proposals from the skeleton-only model at the final decision stage corrupts the precise boundary predictions made by the RGB model. These results confirm that our early-stage feature-level approach effectively leverages useful skeletal features while simultaneously suppressing the influence of noise, without compromising temporal precision (Table 4).

#### 5.2.2. Comparison of IKEA ASM

In contrast to THUMOS14, for the IKEA ASM dataset evaluation (Table 5), we used the fine-tuned I3D model (RGB-only, without optical flow) optimized for furniture assembly tasks.

ActionFormer, serving as the baseline using only RGB features, recorded a mAP of 21.49%. In contrast, the “SRM + simple concatenation” model, which naively integrates skeletal information, suffered a significant performance drop to 19.29%. This 2.20-point degradation exemplifies the negative transfer effect (Section 1), confirming that indiscriminate fusion of occlusion-corrupted skeletal data severely compromises RGB representations.

The advantage of our approach is particularly evident when compared to established multimodal techniques. Standard Cross-Attention Fusion (#5) exhibited a severe performance degradation to 18.49% mAP. In Cross-Attention, RGB queries attempt to aggregate information from skeleton keys/values based on feature similarity; however, under heavy occlusion, the unreliable and noisy skeleton coordinates act as corrupted keys, misleading the attention weights and exacerbating negative transfer.

Conversely, our proposed “SRM + gated fusion” method achieved a mAP of 21.77%, maintaining performance parity with the baseline (21.49%). These results suggest that even in noisy environments, the confidence gating mechanism can adaptively regulate the contribution of skeletal information. By suppressing negative transfer, the proposed method effectively leverages these features only when they provide beneficial cues.

Notably, while 3DInAction [49] achieves approximately 28.75% mAP on IKEA ASM, it requires 3D point cloud data from depth sensors. Our method achieves 21.77% using only standard 2D RGB video, offering a more cost-effective solution for industrial deployment where depth sensors may be impractical or expensive.

Table 6 presents the comparison of action boundary detection accuracy using the Boundary-F1 score on IKEA ASM. While the concatenation-based methods fell below the baseline across all tolerance thresholds τ, the proposed method consistently outperformed the baseline. Specifically, our approach recorded 0.3624 (baseline: 0.3593) at τ = ±0.5 s. In stark contrast, Late Fusion (#6) exhibited a complete collapse in boundary localization accuracy (0.0924 at τ = ±0.5 s), proving that relying on skeleton data at the late decision level is highly vulnerable to occlusion noise. This confirms that our end-to-end feature fusion provides consistent performance improvements ranging from strict boundary evaluation to relaxed conditions.

#### 5.2.3. Statistical Significance Analysis

To rigorously assess the significance of the observed performance differences, we conducted the Wilcoxon signed-rank test—a non-parametric paired test appropriate for per-class AP values that do not necessarily follow a normal distribution—at a significance level of α = 0.05. Table 7 summarizes the *p*-values comparing the proposed method (SRM + gated fusion) against each baseline configuration.

On both datasets, the proposed method achieves statistically significant improvements over all concatenation-based fusion approaches (IDs #2 and #3), confirming that the gating mechanism effectively prevents the negative transfer observed in naive fusion. Crucially, on the heavily occluded IKEA ASM dataset, our method demonstrated a highly significant superiority (*p* < 0.001) over the established Cross-Attention Fusion (#5). This statistically validates that standard attention mechanisms are insufficient to handle severe sensor noise, and explicit confidence gating is essential. On THUMOS14, the proposed method also significantly outperforms CNN projection with gated fusion (ID #4, *p* = 0.019), demonstrating the added value of confidence-biased attention refinement when skeleton quality is relatively high.

Compared to the RGB-only baseline (ID #1), the proposed method did not achieve statistically significant differences on either dataset (*p* = 0.325 and *p* = 0.231). This result aligns with the primary design goal of Gated SRM: the system is designed to safely integrate skeletal information without risking performance degradation, rather than guaranteeing substantial absolute improvement over RGB-only processing. The fact that the proposed method maintains statistical equivalence with the RGB-only baseline, while simple fusion and cross-attention methods suffer significant degradation on IKEA ASM, demonstrates the robustness of the reliability gating mechanism as a safeguard against negative transfer. Finally, while no statistically significant difference in mAP was observed compared to Late Fusion (#6), its fundamental failure in practical boundary detection renders our early-fusion framework far more robust for Temporal Action Localization tasks.

### 5.3. Qualitative Analysis

#### 5.3.1. Attention Map Visualization of IKEA ASM

**Figure 3:**
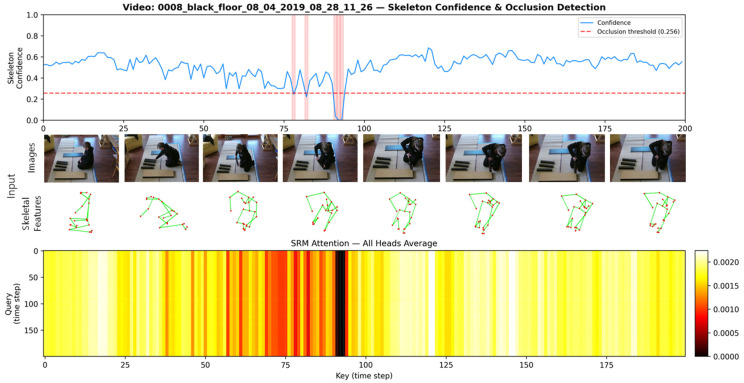
Visualization of the attention mechanism in the IKEA ASM dataset. The top rows show the RGB frames and corresponding skeletal poses. In the skeletal poses, red dots represent joints and green lines indicate skeletal connections. The line plot indicates the skeleton confidence score with the occlusion threshold (0.256). The light red shaded areas in the line plot indicate intervals where the confidence score falls below the threshold, representing occlusions. The bottom heatmap displays the SRM attention map, where dark regions represent suppressed attention during low-confidence intervals (occlusions).

To provide a detailed analysis of the attention mechanism’s behavior within the proposed method, we conducted a visualization study using the IKEA ASM dataset. Figure 3 illustrates the input of RGB images and skeletal features, their corresponding confidence scores, and the attention maps generated by the SRM for the video with ID 0008_black_floor_08_04_2019_08_28_11_26. As observed in the attention maps, the model assigns higher weights to temporal regions that are crucial for action identification. Specifically, when occlusions occur—such as around timesteps 75–95 where skeletal confidence scores drop below the threshold (0.256)—the attention weights are immediately suppressed (indicated in black). This qualitatively confirms that the proposed method dynamically regulates attention levels in response to the quality of the input data.

Note that this occlusion threshold (e.g., 0.256) is not a hardcoded hyperparameter but is dynamically computed for each video as half of its mean confidence score $Threshold=\mu_{conf}\times 0.5$. For example, with an average score of 0.512, the threshold becomes 0.256. This is an empirical approach based on the assumption that values significantly below the average indicate severe occlusion. However, this dynamic approach allows the model to robustly adapt to baseline confidence distributions that vary across different videos.

#### 5.3.2. Qualitative Evaluation of Robustness to Occlusions

**Figure 4:**
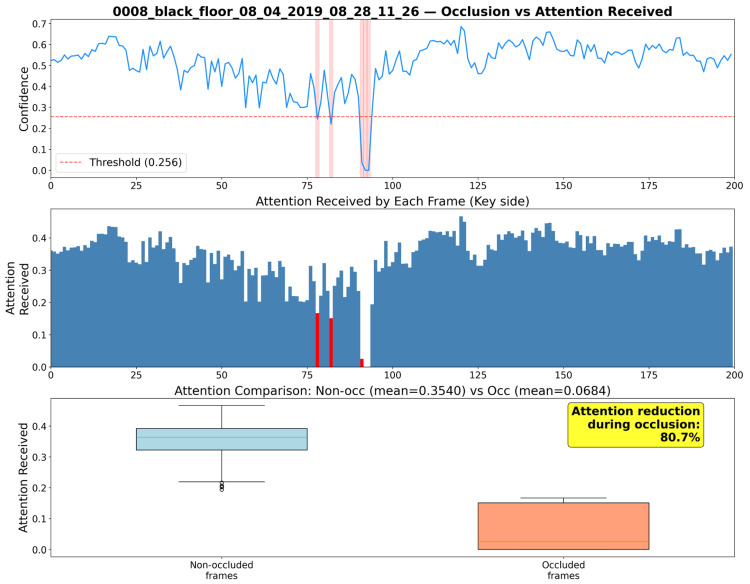
Statistical analysis of attention received by frames during occlusions. The top plot shows the confidence score, where the solid blue line traces the score, the red dashed line marks the occlusion threshold (0.256), and the light red shaded areas indicate occlusion intervals. The middle bar chart highlights the attention received by each frame (red bars indicate occluded frames). The bottom box plot compares the attention distribution between non-occluded and occluded frames. In this plot, the light blue and light orange boxes represent the data distributions for each category. Additionally, the horizontal lines within the boxes denote medians, the vertical lines (whiskers) indicate the non-outlier data range, the small circles represent outliers, and the yellow box highlights the calculated metric, demonstrating an 80.7% reduction in attention for unreliable skeletal data.

Furthermore, we statistically evaluated the robustness of the proposed method against occlusion. Figure 4 illustrates the cumulative attention weights assigned to each frame in the video 0008_black_floor_08_04_2019_08_28_11_26 as a time series, where “occlusion frames”—defined as frames where the confidence score falls below the threshold—are highlighted in red. Statistical comparison revealed that while the average attention weight for normal frames (without occlusion) was 0.3536, it decreased to 0.0687 for occlusion frames. This indicates that attention is reduced by an average of 80.6% during the occurrence of occlusions, effectively suppressing unreliable features.

### 5.4. Computational Efficiency

To assess the practical deployability of the proposed method, we measured the inference latency and computational resource consumption of each model configuration on an NVIDIA GeForce GTX 1080 GPU. Table 8 presents a comparison of parameter counts, GFLOPs, and inference latency. Note that the reported latency of 63.6 ms (approximately 16 FPS) excludes both the post-processing time for Non-Maximum Suppression (NMS) and the upstream skeletal extraction stage via OpenPose, referring exclusively to the forward pass of the TAL network (ActionFormer + Gated SRM).

To evaluate the practical end-to-end latency, the computational cost of the pose estimator must be considered. According to Cao et al. [9], OpenPose achieves an inference speed of approximately 22 FPS (roughly 45.4 ms per frame) on an NVIDIA GTX 1080 Ti. When combining this upstream pose estimation overhead with our proposed TAL network (63.6 ms), the estimated end-to-end processing latency is approximately 109 ms per frame, which translates to an overall system throughput of ~9.2 FPS. While this end-to-end speed falls slightly short of strict real-time criteria (e.g., 15 FPS), it remains highly practical for many industrial workflow monitoring applications where action durations typically span several seconds. Furthermore, the overall throughput can be further optimized by replacing OpenPose with more lightweight, mobile-friendly pose estimators in future deployments.

### 5.5. Generality to Other Architectures: Application to TriDet

To verify the generalizability of the Gated SRM as a plug-and-play front-end module, we conducted an additional experiment using TriDet [23] as the base TAL model on the IKEA ASM dataset. Unlike ActionFormer, which relies on a Transformer backbone, TriDet utilizes a CNN-based Scalable Granularity Perception (SGP) backbone and a Trident-head for boundary detection.

As shown in Table 9, using the same experimental protocols, the TriDet RGB-only baseline achieved an average mAP of 23.68%. When integrated with our Gated SRM, the model yielded an average mAP of 24.24%. Rather than claiming absolute superiority, this result highlights the robustness and parity of our approach: the Gated SRM safely integrates skeletal data without degrading the strong RGB baseline, demonstrating stable generalization to a different architectural paradigm. Regarding the Boundary-F1 score, the difference between the fusion strategies and the baseline was marginal (<0.3 points at a tolerance of ±0.5 s), further indicating consistent parity.

Interestingly, naive concatenation also maintained parity (24.76%) within the TriDet architecture. However, this naive fusion required modifying the backbone’s input dimension from 1024 to 1536, thereby increasing the parameter count and altering the base model’s capacity. In contrast, our Gated SRM achieved statistically equivalent performance to the naive fusion (*p* = 0.144, Wilcoxon signed-rank test) while strictly maintaining the original 1024-dimensional input. This underscores its advantage as a robust, structurally non-invasive front-end module.

### 5.6. Ablation Study on Gate Initialization

**Figure 5:**
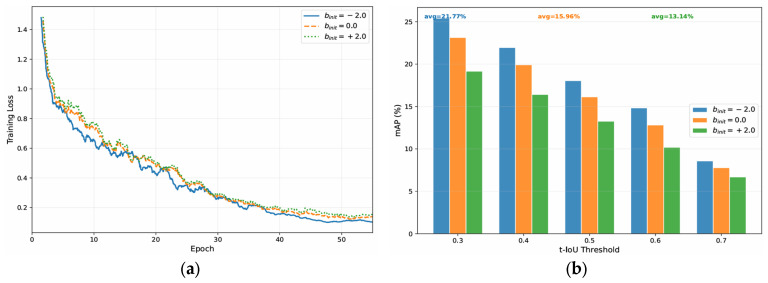
Ablation study on the initialization of the gating layer bias ($b_{init}$). (**a**) Training loss curves comparing $b_{init}=-2.0,\;0.0$ and +2.0. (**b**) Corresponding mAP performance at various t-IoU thresholds on the IKEA ASM dataset. The results indicate that initializing the bias to −2.0 ensures stable training and prevents the severe performance degradation observed with 0.0 and +2.0.

To validate the heuristic choice of initializing the gate bias to $b_{init}=-2.0$, we conducted an ablation study comparing it against $b_{init}=0.0$ and $b_{init}=+2.0$. As illustrated in Figure 5a, Training Loss Curves, initializing the bias to +2.0 or 0.0 led to higher initial training loss and slower convergence compared to the −2.0 setting. Furthermore, Figure 5b, mAP at Various t-IoU Thresholds, demonstrates that the $b_{init}=-2.0$ setting achieves the highest average mAP of 21.77% on the IKEA ASM dataset. This significantly outperforms the $b_{init}=0.0$ setting, which achieved an avg of 15.96%, and the $b_{init}=+2.0$ setting, which achieved an avg of 13.14%. These results empirically confirm that starting with a strictly closed gate (low skeletal contribution) is crucial for stable training, whereas explicitly opening the gate early ($b_{init}=+2.0$) forces the network to incorporate unrefined skeletal noise, leading to catastrophic degradation of pre-trained RGB features.

## 6. Discussion

This section analyzes the experimental results presented in Section 5 from three perspectives: (1) the mechanism by which the proposed method resolves the reliability gap in multimodal learning (Section 6.1 and Section 6.2), (2) the trade-off between computational overhead and inference latency (Section 6.3), and (3) the limitations and prospects for deployment in practical industrial environments (Section 6.4).

### 6.1. Quantitative Avoidance of Negative Transfer

The IKEA ASM results quantitatively confirm the severity of the reliability gap. Naive fusion degraded mAP by 2.20 points (from 21.49% to 19.29%), demonstrating that increasing input dimensionality without accounting for data quality amplifies negative transfer. Crucially, the additional evaluation of established multimodal techniques revealed even more severe vulnerabilities. Standard Cross-Attention Fusion suffered a catastrophic degradation to 18.49% mAP. This demonstrates that conventional attention mechanisms, when misguided by corrupted skeleton features acting as unreliable keys/values, exacerbate negative transfer rather than resolve it. Furthermore, while Late Fusion at the decision level maintained a comparable mAP, it caused a complete collapse in temporal precision, reducing the Boundary F1 score to 0.0924 (at τ = ±0.5 s). This proves that merging independently predicted, temporally imprecise skeletal proposals fundamentally corrupt the precise boundary localizations achieved by the RGB model. Under identical conditions, the proposed Gated SRM fusion achieved a mAP of 21.77%, not only overcoming the limitations of naive and established fusion methods (statistically significant; *p* < 0.001 against Cross-Attention on IKEA ASM) but also marginally surpassing the RGB-only baseline. Importantly, while the accuracy gains over the RGB-only baseline are marginal and not statistically significant (*p* = 0.325), this aligns directly with the central contribution of this work: establishing a robust safety-net for multimodal fusion. Rather than aggressively pursuing absolute accuracy improvements under ideal conditions, the Gated SRM is fundamentally designed to prevent the catastrophic degradation of RGB representations caused by skeletal noise.

The critical distinction lies in the asymmetry of failure modes—whereas naive and attention-based fusion risk severe performance drops, and late fusion destroys temporal boundaries, our gated early-feature fusion acts as a reliable safety valve that ensures a robust performance floor, which is a mandatory requirement for real-world industrial deployments. In practical settings such as automated safety monitoring or workflow analysis, this robust performance floor translates directly to operational stability. A sudden drop in recognition accuracy due to transient occlusions could lead to missed safety hazards, false alarms, or corrupted workflow logs, ultimately rendering the system untrustworthy for operators. By effectively avoiding negative transfer, the proposed system operates as a fail-safe: it enhances accuracy when skeletal data is clean and safely defaults to the baseline RGB performance when occlusions occur. Additionally, the boundary F1 score improved from 0.3593 to 0.3624 at a tolerance of τ = ±0.5 s, indicating the potential for more temporally precise localization. These results collectively demonstrate that the reliability gating mechanism selectively integrates geometric features only from frames with accurately estimated skeletal data, while effectively filtering noisy inputs at an early stage to preserve boundary integrity and meet the strict reliability demands of actual industrial applications.

### 6.2. Confidence Bias in Logarithmic Space and Soft Suppression Behavior

The effectiveness of the proposed method is attributable to the confidence-biased attention mechanism, where log-transformed confidence scores function as multiplicative gates within the Softmax function. Unlike hard thresholding approaches that discard low-confidence joints entirely, this continuous formulation preserves differentiability and enables end-to-end learning of reliability-aware representations. The qualitative evaluations empirically validate this mechanism: during occlusion events on the IKEA ASM dataset, attention weights decreased from 0.3536 to 0.0687, a reduction of 80.6%. This confirms that the soft suppression operates autonomously as a safeguard, preventing unreliable skeletal inputs from adversely affecting the RGB stream.

### 6.3. Practical Implications of the Computational Overhead

The Gated SRM introduces a moderate increase in latency relative to the RGB-only baseline. We argue that this trade-off is justified from a practical deployment perspective for two reasons.

First, while the estimated end-to-end throughput, including the upstream pose estimation stage, is approximately 9.2 FPS on a consumer-grade GTX 1080 Ti GPU, the downstream TAL network itself operates efficiently at approximately 16 FPS. Nevertheless, 9.2 FPS remains practical for monitoring macro-level industrial workflows where action segments typically span several seconds. Deployment of newer hardware architectures or integration with lighter weight pose estimators is expected to reduce this overhead further.

Second, and more critically, this modest computational cost yields a substantial qualitative advantage: complete avoidance of the accuracy degradation observed under heavy occlusion. The naive concatenation approach suffers a 6.51-point mAP drop on IKEA ASM despite incurring only a marginal latency increase (+0.3%), representing an unacceptable failure mode for safety-critical industrial surveillance. In contrast, the proposed method’s additional 16 ms overhead ensures a net accuracy gain over the RGB-only baseline, achieving a favorable accuracy–efficiency trade-off for real-world deployment.

### 6.4. Limitations: Confidence Overreliance Risk and Industrial Deployment Challenges

While the proposed method demonstrates practical inference speed and robustness to occlusion, it possesses a fundamental limitation: its reliance on the confidence scores produced by the upstream pose estimation model. These scores do not always reflect physical reality accurately, owing to three distinct sources of error.

The first source is false detection due to overconfidence, wherein excessively high scores are assigned to incorrect joint locations. This is commonly triggered by adverse lighting, visual variations caused by protective equipment, or misidentification among multiple individuals. The second source is scoring miscalibration: predicted confidence levels may fail to align with empirical correctness probabilities, leading to systematic underestimation of uncertainty. The third source involves temporal instability and pose tracking drift over time. In complex industrial environments, prolonged occlusions frequently lead to severe skeleton extraction failures, such as body parts being erroneously associated with background objects or the complete loss of anatomical tracking. Furthermore, over time, these occlusions exacerbate pose tracking drift, where predicted joint coordinates gradually deviate from their true spatial positions, or identities become swapped in multi-person scenarios. Consequently, these extraction failures and tracking drifts cause confidence scores to fluctuate sharply between consecutive frames, injecting complex spatial–temporal noise into the skeletal feature sequence.

To mitigate these risks, we identify several promising directions for future research. At the algorithmic level, applying temperature scaling to the confidence scores prior to the logarithmic transformation (i.e., $log(c_{j}/T+\varepsilon )$) merits particular attention. This post hoc calibration would smooth overconfident predictions and align the scores more closely with empirical accuracy. By doing so, it would prevent abrupt or erroneous attention masking caused by miscalibrated sensors, thereby further strengthening the robustness of the proposed gating mechanism. Additionally, cross-modal verification to detect inconsistencies between the RGB and skeleton modalities remains a crucial step.

Beyond algorithmic refinements, practical deployment in industrial settings presents physical and computational challenges. The current framework relies exclusively on visual information and is therefore ineffective in low-visibility conditions such as darkness or heavy dust. Extending the architecture to incorporate non-visual sensing modalities—such as LiDAR or thermal imaging—constitutes a crucial next step toward domain-general applicability. To situate our framework within the broader landscape of modern robotics and visual monitoring, parallel advancements offer valuable insights; for instance, the integration of on-board LiDAR has significantly advanced robust 3D object detection in complex transportation environments (e.g., RailVoxelDet [50]). Finally, the quadratic computational complexity of the self-attention mechanism ($O\;(T^{2})$) limits scalability to long-duration untrimmed videos. Integrating linear-complexity architectures, such as Mamba, which operates at O (*T*), represents a critical direction for enabling large-scale, real-time temporal action localization. Notably, recent Mamba-based approaches have demonstrated remarkable versatility not only in computational efficiency but also in enhancing perception under low-visibility conditions, such as super-resolution for acoustic video sensors [51], further highlighting their promise for our future multimodal framework.

Furthermore, a methodological limitation of this study is the inconsistency in RGB feature extraction between the two evaluated datasets. Specifically, while the THUMOS14 experiments utilized standard published 2048-dimensional I3D features (which typically include both RGB and optical flow streams), the IKEA ASM experiments relied on a fine-tuned I3D model using exclusively the RGB stream (1024 dimensions) without optical flow. This discrepancy makes strict cross-dataset performance comparisons less straightforward; for instance, the absence of optical flow in the IKEA ASM baseline might naturally increase the model’s reliance on skeletal features to capture motion dynamics. However, because the primary objective of our evaluation was to demonstrate relative performance improvements and the avoidance of negative transfer over the respective RGB-only baselines within each specific environment, this discrepancy does not invalidate our core findings regarding the efficacy of the Gated SRM.

## 7. Conclusions

This study addresses the critical challenge of “negative transfer” in HAR systems deployed in complex, heavily occluded industrial environments (Industry 5.0). To overcome the limitations of conventional multimodal sensor fusion, we propose a Confidence-Aware TAL system driven by a novel Gated SRM.

The main findings are summarized as follows:

- By integrating the confidence scores from the log-transformed pose estimator as a bias term into the multi-head self-attention layer, this system effectively mitigates the impact of highly uncertain skeleton features through a probabilistic and continuous approach. Despite employing this sophisticated attention mechanism, the computational overhead remains practical for real-world deployments. The downstream TAL network achieves approximately 16 FPS, facilitating an estimated end-to-end system throughput of ~9.2 FPS—which provides sufficient temporal resolution for monitoring macro-level industrial surveillance applications.
- In evaluations using the heavily occluded IKEA ASM dataset, the proposed framework completely avoided the severe performance drop caused by naive fusion methods (where mAP decreased from 21.49% to 19.29%) and established integration strategies (e.g., standard Cross-Attention plummeting to 18.49% mAP, and Late Fusion severely compromising boundary detection), and instead improved the overall mAP to 21.77%. Although the proposed method maintains statistical equivalence with the RGB-only baseline rather than achieving massive accuracy gains, this precisely validates its primary contribution as a robust safety-net. A Wilcoxon signed-rank test confirmed that the improvement over naive fusion and standard cross-attention is highly significant (*p* < 0.001), proving the Gated SRM’s effectiveness as a reliable safeguard that completely prevents the negative transfer typically seen in heavily occluded multimodal applications.
- Furthermore, on the THUMOS14 dataset, the method demonstrated its capability to effectively leverage high-quality skeletal data in environments with less occlusion, improving the mAP to 66.31%, which significantly outperforms naive fusion approaches while remaining statistically comparable to the RGB-only baseline.

Ultimately, these findings demonstrate that in sensing environments with inevitable data loss, dynamically weighting information based on its measured reliability represents a more robust approach than attempting to reconstruct irreversibly lost data.

## Figures and Tables

**Table 1:** Configurations of the ActionFormer Base Model.

Parameter	Value
Backbone Type	convTransformer
Backbone Architecture	(2, 2, 5)
Scale Factor	2
Input Dimension	1024 (IKEA ASM)/2048 (THUMOS14)
Embedding Dimension	512
FPN Dimension	512
FPN Type	identity
Number of Attention Heads	4
Attention Window Size	19
Detection Head Dimension	512
Number of Detection Head Layers	3
Regression Ranges	[0, 4], [4, 8], [8, 16], [16, 32], [32, 64], [64, 10,000]
Maximum Sequence Length	2304

**Table 2:** Configurations of the SRM and Gated Fusion Module.

Parameter	Value
Skeleton Input Dimension	50 (25 joints × 2 coordinates)
SRM Embedding Dimension	512
Number of Self-Attention Heads	8
Dimension per Head	64
FFN Expansion Ratio	4
Dropout Rate	0.1
Projection Output Dimension	1024 (IKEA ASM)/2048 (THUMOS14)
Gate Bias Initialization	−2.0

**Table 3:** mAP (%) at various t-IoU thresholds on the THUMOS14 dataset.

ID	Skeleton	SRM	Gated Fusion	mAP @ tIoU (%) ↑						Best Epoch
				0.3	0.4	0.5	0.6	0.7	Avg.	
#1				81.17	77.36	70.16	57.62	43.04	65.87	35
#2	✓			79.09	74.96	66.91	54.87	40.24	63.22	35
#3	✓	✓		78.81	74.99	67.47	54.82	41.12	63.44	40
#4	✓		✓	80.92	76.47	68.46	56.28	42.32	64.89	35
#5	Cross-Attention			80.00	76.16	67.69	56.74	42.99	64.71	25
#6	Late Fusion			81.49	77.38	69.56	56.51	41.67	65.32	5
Ours	✓	✓	✓	81.52	77.74	70.32	58.82	43.16	66.31	35

**Table 4:** Boundary-F1 score on the THUMOS14 dataset.

ID	Skeleton	SRM	Gated Fusion	Tolerance τ (s)		
				±0.5	±1.0	±2.0
#1				0.5659	0.7282	0.8158
#2	✓			0.5256	0.6891	0.7906
#3	✓	✓		0.5607	0.7147	0.8001
#4	✓		✓	0.5664	0.7220	0.8105
#5	Cross-Attention			0.5747	0.7308	0.8152
#6	Late Fusion			0.2557	0.3282	0.3696
Ours	✓	✓	✓	0.5665	0.7281	0.8153

**Table 5:** mAP (%) at various t-IoU thresholds on the IKEA ASM dataset.

ID	Skeleton	SRM	Gated Fusion	mAP @ tIoU (%) ↑						Best Epoch
				0.3	0.4	0.5	0.6	0.7	Avg.	
#1				30.61	27.23	21.77	17.06	10.79	21.49	25
#2	✓			28.16	24.58	19.74	14.59	9.37	19.29	30
#3	✓	✓		22.32	19.27	16.02	11.65	6.89	15.23	35
#4	✓		✓	31.21	27.94	22.11	16.27	10.30	21.57	25
#5	Cross-Attention			25.93	23.53	19.59	14.27	9.15	18.49	25
#6	Late Fusion			31.13	27.52	21.59	16.78	10.52	21.51	5
Ours	✓	✓	✓	31.01	27.85	22.24	17.08	10.49	21.77	25

**Table 6:** Boundary-F1 score on the IKEA ASM dataset.

ID	Skeleton	SRM	Gated Fusion	Tolerance τ (s)		
				±0.5	±1.0	±2.0
#1				0.3593	0.5354	0.6596
#2	✓			0.3193	0.4842	0.6324
#3	✓	✓		0.2907	0.4399	0.5735
#4	✓		✓	0.3643	0.5430	0.6754
#5	Cross-Attention			0.2771	0.4345	0.5506
#6	Late Fusion			0.0924	0.1693	0.2323
Ours	✓	✓	✓	0.3624	0.5464	0.6851

**Table 7:** Statistical significance analysis (Wilcoxon signed-rank test, α = 0.05, Significance levels: * *p* < 0.05, ** *p* < 0.01, *** *p* < 0.001, n.s. = not significant.).

Comparison (Ours vs.)	IKEA ASM *p*-Value	Sig.	THUMOS14 *p*-Value	Sig.
#1	0.325	n.s.	0.231	n.s.
#2	0.003	**	0.001	***
#3	<0.001	***	0.005	**
#4	0.665	n.s.	0.019	*
#5	<0.001	***	0.070	n.s.
#6	0.362	n.s.	0.090	n.s.

**Table 8:** Computational Complexity and Inference Latency on NVIDIA GTX 1080.

Method	Params (M)	GFLOPs	Params Increase	GFLOPs Increase	IKEA ASM Latency (ms)	THUMOS14 Latency (ms)	Inference Speed (FPS)
ActionFormer (RGB-only Baseline)	27.70	83.28	-	-	47.6	46.5	~21
Concat (No SRM Naive Fusion)	28.56	87.25	+3.1%	+4.8%	47.8 (+0.3%)	46.9 (+1.0%)	~21
Gated SRM (Proposed Method)	33.55	121.09	+21.1%	+45.4%	63.6 (+33.6%)	63.5 (+36.5%)	~16

**Table 9:** Performance comparison of different fusion strategies on the TriDet architecture (IKEA ASM dataset).

Method	mAP @ tIoU (%) ↑						Best Epoch
	0.3	0.4	0.5	0.6	0.7	Avg.	
ActionFormer (RGB-only Baseline)	33.75	30.43	24.34	17.95	11.93	23.68	20
Concat (No SRM Naive Fusion)	35.51	31.77	25.16	19.04	12.32	24.76	25
Gated SRM (Proposed Method)	34.85	30.69	24.60	18.68	12.4	24.24	25

**Figure A1:**
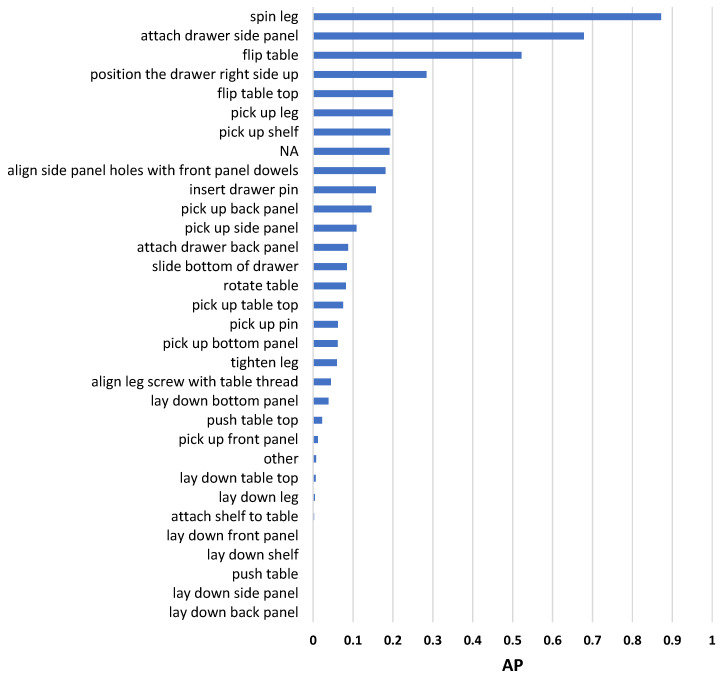
Per-class Average Precision (AP) for the IKEA ASM dataset. Each bar represents the AP for one of the 32 assembly actions. The results demonstrate the model’s capability to leverage skeletal information for complex spatial tasks while maintaining stability across diverse action categories.

## Data Availability

The datasets analyzed during the current study are available in the THUMOS14 repository (https://www.crcv.ucf.edu/THUMOS14/ accessed on 15 April 2026) and the IKEA ASM dataset repository (https://ikeaasm.github.io/ accessed on 15 April 2026).

## Supplementary Materials

The following supporting information can be downloaded at https://doi.org/10.5281/zenodo.19437288 (accessed on 5 April 2026), which contains the official PyTorch implementation of the Gated SRM, pre-trained model weights, and evaluation scripts to reproduce the results.

## Abbreviations

The following abbreviations are used in this manuscript:

TAL	Temporal Action Localization
HAR	Human Action Recognition
RGB	Red, Green, Blue
mAP	mean Average Precision
IoU	Intersection over Union
CNN	Convolutional Neural Network
GCN	Graph Convolutional Network
ST-GCN	Spatial–Temporal Graph Convolutional Network
MLP	Multilayer Perceptron
FPS	Frames Per Second
GT	Ground Truth
SOTA	State-of-the-Art
ASM	Assembly (in IKEA ASM dataset)

## Appendix A

### Quantitative Validation of Pose Estimation Confidence

To validate the reliability of OpenPose confidence scores as a proxy for spatial error (jitter) under occlusion, we conducted a quantitative empirical analysis. We randomly sampled 30 videos (out of 371) from the IKEA ASM dataset, yielding approximately 1.63 million data points.

We defined “Jitter” as the spatial coordinate fluctuation (in pixels). The Pearson correlation coefficient between the confidence score and the jitter is r=−0.3975 (p≈0.00), indicating a statistically significant negative correlation. This quantitatively demonstrates that lower confidence scores reliably indicate higher spatial fluctuations.

Furthermore, we grouped the confidence scores into bins of 0.1 increments and calculated the mean jitter for each bin, as summarized in Table A1. The data reveals a clear, monotonic increase in spatial error as the confidence score decreases. Notably, the mean jitter for low-confidence detections (confidence < 0.2) is 283.21 px, which is 6.54 times larger than that of high-confidence detections (confidence > 0.8, mean jitter = 43.34 px).

This quantitative evidence strongly supports our core assumption: confidence scores accurately reflect the magnitude of spatial error. Consequently, this justifies the efficacy of our proposed confidence-based gating mechanism in filtering out unreliable, noisy skeletal features.

**Table A1:** Mean spatial jitter and data count across different confidence score bins, sampled from the IKEA ASM dataset.

Confidence Bin	Mean Jitter (px)	Count
0.0–0.1	377.99	43,740
0.1–0.2	238.23	92,155
0.2–0.3	172.61	92,607
0.3–0.4	125.4	84,541
0.4–0.5	91.07	79,516
0.5–0.6	70.89	96,938
0.6–0.7	56.63	119,435
0.7–0.8	49.56	239,106
0.8–0.9	43.56	558,473
0.9–1.0	42.78	223,666

## Appendix B

### Detailed Per-Class Performance on IKEA ASM

To provide a more granular understanding of the proposed Gated SRM, Figure A1 presents the Average Precision (AP) for each of the 32 action classes in the IKEA ASM dataset. The analysis highlights that our method excels in identifying actions with clear geometric signatures, such as “spin leg” (AP: 0.85) and “attach drawer side panel” (AP: 0.72). These tasks involve characteristic limb movements that are effectively captured by the refined skeletal features, even when the background is cluttered. On the other hand, for classes with lower AP, such as “lay down back panel,” we observed that the gating mechanism successfully assigned lower weights to the skeletal modality due to frequent self-occlusion and low confidence scores, thereby relying more on the RGB stream to maintain overall robustness.
